# Giant microwave absorption in fine powders of superconductors

**DOI:** 10.1038/s41598-018-29750-7

**Published:** 2018-07-31

**Authors:** G. Csősz, B. G. Márkus, A. Jánossy, N. M. Nemes, F. Murányi, G. Klupp, K. Kamarás, V. G. Kogan, S. L. Bud’ko, P. C. Canfield, F. Simon

**Affiliations:** 1 Department of Physics, Budapest University of Technology and Economics and MTA-BME Lendület Spintronics Research Group (PROSPIN), POBox 91, H-1521 Budapest, Hungary; 2 GFMC, Unidad Asociada ICMM-CSIC “Laboratorio de Heteroestructuras con Aplicacion en Espintronica”, Departamento de Fisica de Materiales Universidad Complutense de Madrid, 28040 Madrid, Spain; 3 Mettler-Toledo GmbH, Heuwinkelstrasse 3., CH-8606 Nänikon, Switzerland; 4 Institute for Solid State Physics and Optics, Wigner Research Centre for Physics, Hungarian Academy of Sciences, P.O. Box 49, H-1525 Budapest, Hungary; 5 Ames Laboratory, U.S. Department of Energy and Department of Physics and Astronomy, Iowa State University, Ames, Iowa 50011 USA

## Abstract

Enhanced microwave absorption, larger than that in the normal state, is observed in fine grains of type-II superconductors (MgB_2_ and K_3_C_60_) for magnetic fields as small as a few % of the upper critical field. The effect is predicted by the theory of vortex motion in type-II superconductors, however its direct observation has been elusive due to skin-depth limitations; conventional microwave absorption studies employ larger samples where the microwave magnetic field exclusion significantly lowers the absorption. We show that the enhancement is observable in grains smaller than the penetration depth. A quantitative analysis on K_3_C_60_ in the framework of the Coffey–Clem (CC) theory explains well the temperature dependence of the microwave absorption and also allows to determine the vortex pinning force constant.

## Introduction

Electrodynamics of superconductors remains an intensively studied field^1^ due to the wealth of attainable fundamental information, including the nature of pairing mechanism and the coupling strength, and also due to the technological importance of these materials. In addition, recent studies focus on novel topological phases including superconductors^2^. As an example, observation of the conductivity coherence peak in conventional superconductors^3^ (Nb and Pb) and its absence in high-*T*_c_ materials^4^ pointed to a BCS mechanism in the former and it was an early indication of non-BCS superconductivity in the latter compounds. Concerning applications, the DC electrodynamic properties in the mixed state of type-II superconductors determine the utility (e.g. loss, permanent field homogeneity, and stability) in superconducting solenoid coils that are widely used in superconducting particle acceleration, solid state spectroscopy, or medical imaging. The AC electrodynamic properties are relevant for applications including e.g. power handling, sound and electromagnetic field detection^5–7^, superconducting microwave resonators^8^, and in microwave absorbers^9^.

The frequency dependent conductivity of superconductors, $\tilde{\sigma }={\sigma }_{1}+i{\sigma }_{2}$, is well known for both BCS (i.e. weak-coupled *s*-wave pairing) and non-BCS superconductors (including strongly coupled *s*-wave and non *s*-wave superconductors) in the absence of magnetic field, *B* = 0. At zero temperature, *T* = 0, the real part, *σ*_1_(*ω*), is a delta function at *ω* = 0 followed by *σ*_1_(*ω*) = 0 until the gap edge at *ω*_g_ = 2Δ/ℏ (ref.^10^) (usually at 0.1–10 THz). According to the Ferrell–Glover–Tinkham (FGT) sum rule^11,12^, the spectral weight of the delta function comes from states which are gapped below *ω*_g_ (the sum rule is discussed in depth in the Supplementary Material). The technologically important radio frequency range spans 9 orders of magnitude in superconductors (from 10 kHz up to 1 THz) with similar characteristic properties, thus measurements in the microwave range (1–100 GHz) are representative.

Conductivity in finite magnetic fields for the mixed state in type-II superconductors was first described by the Bardeen–Stephen (BS) model^13^ for the viscous motion of vortices. This was later improved by the Coffey–Clem theory (CC) in a series of seminal papers^14–19^, which also includes the effect of pinning force on the vortex motion. The most important prediction of the BS model is a finite *σ*_1_ conductivity at *ω* = 0. However, it is less well-known that the FGT sum rule implies a non-zero *σ*_1_ that is *larger* than in the normal state for *ω* < *ω*_g_. Observation of this effect has been elusive as most contributions study the surface impedance on polycrystalline^3^, compacted powder pellet, or thin film samples^20,21^. Surface impedance studies have the advantage that sample geometry is well defined, however the effects of *σ*_1_ and *σ*_2_ are inevitably intermixed in this type of measurements. Given that *σ*_2_ is orders of magnitude larger than *σ*_1_ in the superconducting state (due to the small value of the penetration depth, *λ*, with respect to sample thickness), the surface impedance measurement is less sensitive to changes in *σ*_1_ (refs^22–26^).

The effects of *σ*_1_ and *σ*_2_ are decoupled for fine grains; for a sample placed in a microwave cavity, the loss is due to *σ*_1_, whereas the resonance shift is due to *σ*_2_ (refs^27–29^). Therefore such samples provide a unique opportunity to test the predictions of the CC theory on *σ*_1,2_(*T*, *B*). The enigmatic and yet unexplained increase of the electron spin resonance signal in superconductors right below *T*_c_ (refs^30,31^) also highlights the need to study further the electromagnetic absorption in superconductors.

This motivated us to revisit the microwave conductivity (at about 10 GHz) in the MgB_2_ and K_3_C_60_ superconductors as a function of *T* and *B*. We observe an excess microwave loss (or microwave absorption) in small magnetic fields (as low as a few % of the upper critical field, *B*_c2_) for a sample consisting of well-separated fine grains (typical size is a few micrometers). The excess microwave absorption is not observable in a single crystal sample. A quantitative analysis is provided for K_3_C_60_, which is a one-gap, cubic superconductor with well known magneto-transport properties^32^, whereas MgB_2_ is a multi-band superconductor with strongly anisotropic *B*_c2_ (refs^33,34^), thus application of the CC model is less straightforward.

## Methods and Experimental

We studied fine powder MgB_2_ samples identical to batches in refs^35^ and^30^. Single crystal and powder K_3_C_60_ samples were prepared by the conventional K intercalation method; the crystal sample was from the same batch as in ref.^36^. The starting fullerene powder material contains grains with a size of a few micrometers that is retained in the K doped material according to literature studies^37–39^. The powder samples were further ground together with non-conducting SnO_2_ powder to prevent conducting links between the grains. Samples were sealed in quartz ampules under low pressure helium. Microwave properties were measured with the cavity perturbation method^27,28^ as a function of temperature, *T*, and in various static magnetic fields, *B*, inside a superconducting solenoid, with zero-field cooling. Zero field measurements (besides the Earth’s magnetic field) were made in another cryostat without a magnet field solenoid to avoid trapped flux (which may amount to 10–20 mT). The unloaded copper cavity has a quality factor, *Q*_0_ ~ 10,000 and a resonance frequency, *f*_0_ ~ 11.2 GHz, whose temperature dependence is taken into account. The samples were placed in the node of the microwave (or *rf*) electric field and maximum of the microwave magnetic field inside the TE011 cavity, which is the appropriate geometry to study minute changes in the conductivity^29^. The *rf* magnetic field is parallel to the DC field of the solenoid, which yields the largest vortex motion induced absorption according to the CC theory^16^. Measurement^40^ of the quality factor, *Q*, and the cavity resonance frequency, *f* yields the loss: ${\rm{\Delta }}(\frac{1}{2Q})=\frac{1}{2Q}-\frac{1}{2{Q}_{0}}$ and cavity shift: Δ*f*/*f*_0_ = (*f* − *f*_0_)/*f*_0_.

## Results

**Figure 1:**
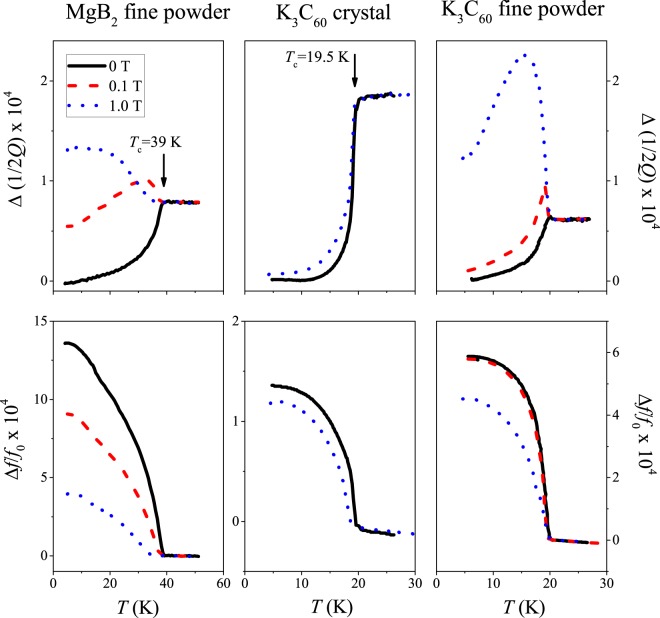
Temperature dependent cavity loss, ${\rm{\Delta }}(\frac{1}{2Q})$, and cavity shift, Δ*f*/*f*_0_ for a fine powder of MgB_2_ and for the single crystal and powder K_3_C_60_ samples. Two magnetic field data are shown for the crystal (0 and 1 T) and three for the powder samples (0, 0.1, and 1 T). Note that the cavity loss changes significantly for the powder sample in contrast to the single crystal sample. Note the different scales for the Δ*f*/*f*_0_ data.

Figure 1 shows the microwave cavity loss and cavity shift for a fine powder of MgB_2_ and for two kinds of K_3_C_60_ samples: a single crystal and a fine powder as a function of temperature and for a few magnetic field values. The microwave loss decreases rapidly below *T*_c_ in zero magnetic field as expected for superconductors. The most important observation is that the microwave loss becomes significant for a magnetic field as small as 0.1 T for the fine powder samples, whereas even 1 T has little effect on the microwave absorption for the single crystal K_3_C_60_ sample. In fact, we observe a *giant*, about a factor 3 times larger, microwave absorption below *T*_c_ than in the normal state. This striking difference between the crystal and fine grain samples is clearly demonstrated for K_3_C_60_ where measurements on both kinds of samples are shown. For MgB_2_, microwave measurements on compacted samples (or surface impedance measurements) supports this observation as therein no enhanced microwave absorption was observed^41–46^. While the absorption becomes significant for the fine powder samples at *B* = 0.1 T, the shift changes less, which means that the overall superconducting characteristics of the sample are maintained.

We believe that the enhanced microwave absorption is an ubiquitous property of fine powders of type-II superconductors. However, we cannot quantitatively discuss this effect for MgB_2_ due to the multi-band superconductivity^47,48^ and the significant *B*_c2_ anisotropy; *B*_c2_ at 0 K is ~2 T and ~16 T for $B\parallel (c)$ and $B\parallel (a,b)$, respectively (refs^34,35,49^). We therefore focus on K_3_C_60_ in the following. The enhanced microwave loss appears progressively with increasing magnetic field (additional data are shown in the Supplementary Material).

We also show the *B* = 0.1 T data for the powder sample (*B* ≈ 0.002 × *B*_c2_) in Fig. 1; they show a peak in the microwave loss right below *T*_c_ followed by a gradual decrease. The zero magnetic field data also shows a small peak (invisible at the scale of Fig. 1) for the powder sample (shown in the Supplementary Material). This small peak is not due to magnetic field and is most probably a tiny conductivity coherence peak (the analogue of the Hebel–Slichter peak^50^) which is known to be strongly suppressed by strong-coupling effects in alkali fullerides^51,52^. While the presence of a coherence peak itself is an interesting physical phenomenon^3,53^, it is not relevant for the present discussion.

The fact that the enhanced microwave absorption occurs with the application of the magnetic field hints at a flux motion related phenomenon that is discussed in the framework of the CC theory. The microwave absorption peak occurs above the irreversibility line, i.e. it is related to the physical behavior of the vortex-fluid state; for K_3_C_60_ *T*_irr_(*B* = 0.1 T) ≈ 15 K and *T*_irr_(*B* = 1 T) < 5 K (ref.^54^).

The strong dependence on the sample morphology is also discussed below. Superconducting fullerides are type-II ($\lambda \gg \xi$) and have a short mean free path^32^ i.e. they can be described in the local electrodynamics limit as opposed to the non-local (or Pippard) limit, which simplifies the discussion.

## Discussion

### Conductivity in the superconducting state

The phenomenological CC theory^14–19^ is based on a two-fluid model and considers the motion of vortices due to the exciting electromagnetic field in the presence of a viscous background (described by the viscous drag coefficient, *η*) and a restoring force (described by an effective pinning force constant, *κ*_p_).

The viscous drag was introduced in the Bardeen–Stephen theory^13^ and is determined by the superconducting parameters^10,55^: $\eta (T)=\frac{{{\rm{\Phi }}}_{0}{B}_{{\rm{c}}2}(T)}{{\rho }_{{\rm{n}}}(T)}$. The value of *κ*_p_ is unknown and only an upper limit can be estimated from thermodynamic considerations^55–57^ for a “perfect pinning center”, i.e. a hollow cylinder with a diameter of about the coherence length, *d* ≈ *ξ*. The condensation energy gain per unit length from placing a vortex in this cylinder is about ${d}^{2}{B}_{{\rm{c}}}^{2}\mathrm{/2}{\mu }_{0}$, where the square of the thermodynamic critical field is ${B}_{{\rm{c}}}^{2}={B}_{{\rm{c}}1}{B}_{{\rm{c}}2}/\,\mathrm{ln}(\frac{\lambda }{\xi })$. This leads to:

1 $${\kappa }_{{\rm{p}},{\rm{\max }}}=\frac{{B}_{{\rm{c}}}^{2}}{2{\mu }_{0}}.$$

For a weaker pinning center, *κ*_p_ can be significantly lower and in the bulk of a perfect superconductor, it would be zero.

The CC theory introduces the concept of the complex penetration depth, $\tilde{\lambda }$:

2 $${\tilde{\lambda }}^{2}=\frac{{\lambda }^{2}+({\rm{i}}\mathrm{/2)}{\tilde{\delta }}_{{\rm{vc}}}^{2}}{1-2{\rm{i}}{\lambda }^{2}/{\tilde{\delta }}_{{\rm{nf}}}^{2}},$$

where ${\tilde{\delta }}_{{\rm{nf}}}$ is the normal fluid skin depth, *λ* is the usual (real) penetration depth and ${\tilde{\delta }}_{{\rm{vc}}}$ is the complex effective skin depth^19^. The latter quantity is zero for *B* = 0 and becomes finite in the mixed state when vortex motion is present. $\tilde{<mml:mpadded xmlns:xlink="http://www.w3.org/1999/xlink" voffset="0">\lambda</mml:mpadded>}$ is related to the complex conductivity by $\tilde{\sigma }=i/{\mu }_{0}\omega {\tilde{<mml:mpadded xmlns:xlink="http://www.w3.org/1999/xlink" voffset="0">\lambda</mml:mpadded>}}^{2}$. Note that for *B* = 0 (i.e. when ${\tilde{\delta }}_{{\rm{vc}}}^{2}=0$), we obtain ${\tilde{<mml:mpadded xmlns:xlink="http://www.w3.org/1999/xlink" voffset="0">\lambda</mml:mpadded>}}^{2}(T=0)={\lambda }^{2}$ and ${\tilde{<mml:mpadded xmlns:xlink="http://www.w3.org/1999/xlink" voffset="0">\lambda</mml:mpadded>}}^{2}(T={T}_{{\rm{c}}})={\rm{i}}{\delta }_{{\rm{n}}}^{2}/2$ as expected. The CC theory yields the temperature and magnetic field dependent $\tilde{\sigma }$ using explicit expressions for *λ*(*T*), *δ*_nf_(*T*), *B*_c2_(*T*). We have implemented the calculation (details are given in the Supplementary Material) and validated our calculations by comparing the results to that published in ref.^19^.

**Figure 2:**
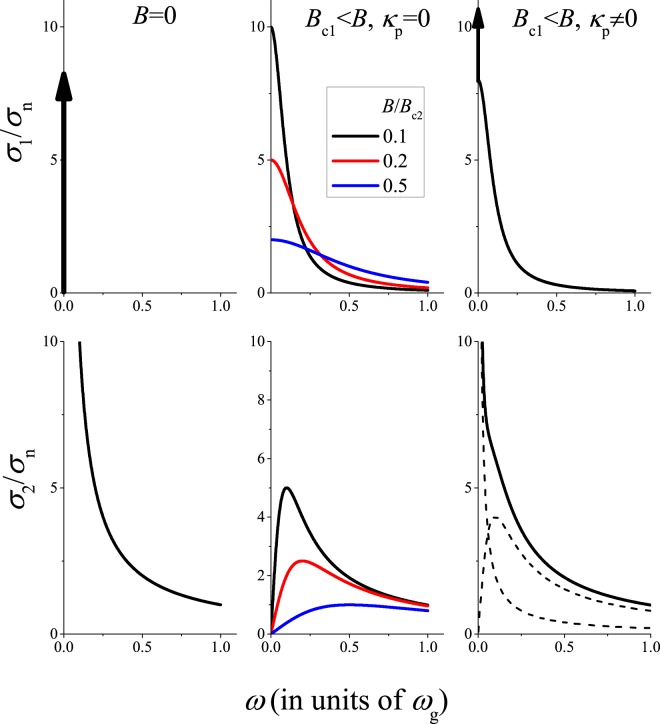
Illustration of $\tilde{\sigma }(\omega )$ in superconductors for i) *B* = 0, ii) for finite fields (*B* > *B*_c1_) with *κ*_p_ = 0 (the Bardeen–Stephen case), and iii) for *B* ≠ 0 and a finite *κ*_p_ (the case of the CC theory). Conductivity above the superconducting gap, *ω*_g_, is not shown. The spectral weight in the delta function is preserved for *B* ≠ 0. Note that for *κ*_p_ ≠ 0, the conductivity appears as if it were a sum of $\tilde{\sigma }$’s for the *B* = 0 and the BS flux-flow regimes (shown with dashed curves). Of the two components, the *σ*_1_ ∝ *δ*(*ω*) and *σ*_2_ ∝ 1/*ω* is due to vortex pinning.

Here, we discuss qualitatively the predictions of the CC theory and some typical cases are shown in Fig. 2. In superconductors, at *B* = 0 the carrier spectral weight below *ω*_g_ collapses into the ${\sigma }_{1}=\frac{\pi }{2{\mu }_{0}{\lambda }^{2}}\delta (\omega )$ function according to the Ferrell–Glover–Tinkham (FGT) sum rule^11,12^. The Kramers–Kronig relation dictates that *σ*_2_ = 1/*μ*_0_*ωλ*^2^. Without vortex pinning, the Meissner state is destroyed for *B* > *B*_c1_ and the Bardeen–Stephen theory gives $\sigma =\frac{{B}_{{\rm{c}}2}}{B}\frac{{\sigma }_{{\rm{n}}}}{1+{\rm{i}}\frac{\omega }{{\omega }_{{\rm{c}}}}}$. It is worth noting that this result is formally analogous to the AC Drude model as the underlying equation of motion (of electrons or vortices) is the same. Here, we introduced a cut-off frequency ${\omega }_{{\rm{c}}}=\frac{B}{{\mu }_{0}{\lambda }^{2}{B}_{{\rm{c2}}}{\sigma }_{{\rm{n}}}}$. Clearly, *σ*_1_ can be larger than *σ*_n_ for *ω* < *ω*_c_.

In the presence of vortex pinning, the CC theory predicts that $\tilde{\sigma }$ is characterized by a mixture of the unperturbed superconducting behavior and that of the Bardeen–Stephen theory with a shared spectral weight which depends on the pinning force constant. Pinning reduces the effect of the vortex flow on *σ*_1_. The enhanced *σ*_1_(*ω*) AC conductivity (as compared to the normal state) is a direct consequence of the FGT sum rule for a finite magnetic field. It allows to estimate the maximum possible value of the enhancement as *σ*_1, max_(*ω*) ≈ *σ*_n_ × *ω*_g_/*ω*, that would be realized at *T* = 0 in the absence of pinning. E.g. for K_3_C_60_ and *ω*/2*π* = 10 GHz we obtain *σ*_1, max_(*ω*) ≈ 140*σ*_n_.

The CC theory allows to quantitatively analyze the conductivity in K_3_C_60_. The requirement of $B\gg {B}_{{\rm{c1}}}$ is satisfied for our magnetic fields of 0.1 … 1 T as *B*_c1_ ~ 10 mT. The CC theory was developed for a superconductor which occupies the total half space. We show in the Supplementary Material that it can be applied for a spherical sample which approximates well finite sized grains containing at least a few hundred/thousand vortices. In addition, the static and *rf* magnetic fields are parallel in our experiment, which is the standard case for the applicability of the CC theory. Albeit we cannot quantitatively consider the effect of the small particle size on the magnetic properties, we believe that neither the surface barriers (also known as Bean-Livingstone barriers^58^) nor the so-called geometrical barriers^59^ affect considerably the applicability of the CC theory. The argument is that both types of barriers would affect the overall number of the vortices under the applied DC magnetic field (or the *B* value where vortices appear) but not the overall vortex dynamics under the application of the small AC magnetic field, which is the primary reason for the observed microwave absorption.

**Figure 3:**
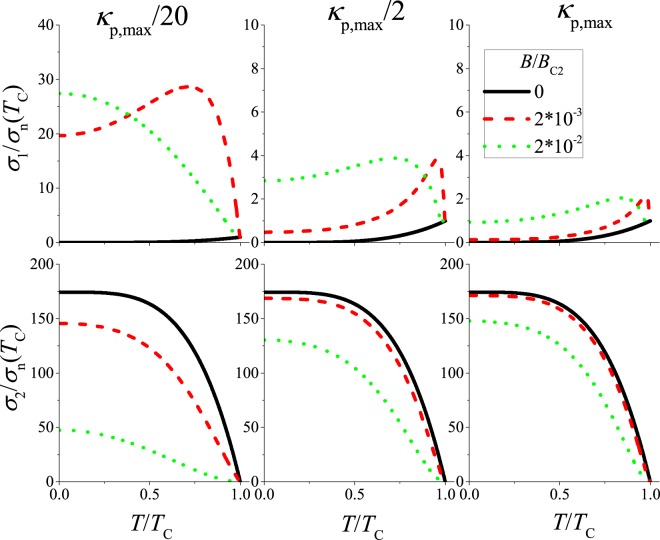
Calculated real part and imaginary part of complex *rf* conductivity vs the reduced temperature for different values of the pinning force constant, *κ*_p_. The conductivity values are normalized by the normal state conductivity at the critical temperature. The large value of *σ*_2_(*T* = 0)/*σ*_n_(*T*_c_) is due to a large (*δ*_n_/*λ*)^2^. Note also the different scales for the *σ*_1_ values.

In Fig. 3, the calculated conductivity is plotted versus the reduced temperature for different force constants, *κ*_p_, with the parameters *ξ*_0_ = 3 nm (which corresponds to *B*_c2_(0 K) = 37.5 T), *λ*_0_ = 440 nm, and *ρ*_n_(*T*_c_) = 2.95 Ωm, the mean values of the corresponding literature parameters for K_3_C_60_ (refs^32,37–39,60–62^), which are detailed in Table 1. The pinning force constant is given in the figure with respect to ${\kappa }_{{\rm{p}},{\rm{\max }}}=3.84\cdot {10}^{4}\,{\rm{N}}/{{\rm{m}}}^{2}$ according to Eq. (1). The figure indicates that *κ*_p_ strongly affects the magnetic field dependence of $\tilde{\sigma }$.

**Table 1:** Transport and magnetic parameters of the K_3_C_60_ superconductor: the superconducting transition temperature, *T*_c_; the normal state resistivity at *T*_c_, *ρ*_n_; the normal state skin depth, *δ*_n_; the coherence length at *T* = 0, *ξ*_0_; and the magnetic field penetration depth at *T* = 0, *λ*_0_.

Property	Value	Refs
*T*_c_	19.5 K	^32^
*ρ*_n_(*T*_c_)	$1.8\cdot {10}^{-6},\,4.1\cdot {10}^{-6}$ Ωm	^60, 61^
*δ*_n_(11.1 GHz)	9.7, 6.4 *μ*m	
*ξ*_0_	2.6, 3.4 nm	^37, 38^
*λ*_0_	240, 480, 600 nm	^38, 39, 62^

The tabulated *ξ*_0_ values correspond to an upper critical field, *B*_c2_ at *T* = 0 of 49 and 28 T, respectively.

### Analysis of the experimental data

The sample morphology greatly affects the relation between the material conductivity, $\tilde{\sigma }$, and the microwave parameters, the loss and shift. Two limiting cases are known. 1) The sample is large and the field penetrates into a limited distance from the surface. This approximates the measurement in the large K_3_C_60_ single crystal. 2) The sample is a small sphere with radius comparable to the penetration depth. This approximates the K_3_C_60_ sample of well divided small grains. We discuss that the experimental observations for the crystal and fine powder K_3_C_60_ are explained well by these two regimes.

In the first case, when the *rf* field penetrates in the skin depth only (known as the skin limit), the following equation holds between the microwave measurement parameters and the material quantities^28^:

3 $$\frac{{\rm{\Delta }}f}{{f}_{{\rm{0}}}}-{\rm{i}}{\rm{\Delta }}(\frac{1}{2Q})=-\,{\rm{i}}\nu {\mu }_{0}\omega \sqrt{-\,{\tilde{<mml:mpadded xmlns:xlink="http://www.w3.org/1999/xlink" voffset="0">\lambda</mml:mpadded>}}^{2}},$$

where the complex penetration depth, $\tilde{<mml:mpadded xmlns:xlink="http://www.w3.org/1999/xlink" voffset="0">\lambda</mml:mpadded>}$, is related to the conductivity as ${\tilde{<mml:mpadded xmlns:xlink="http://www.w3.org/1999/xlink" voffset="0">\lambda</mml:mpadded>}}^{2}={\rm{i}}{({\mu }_{{\rm{0}}}\omega \tilde{\sigma })}^{-1}$. The dimensionless $\nu \ll 1$ is the so-called resonator constant^3^ and it depends on the sample surface relative to that of the cavity.

**Figure 4:**
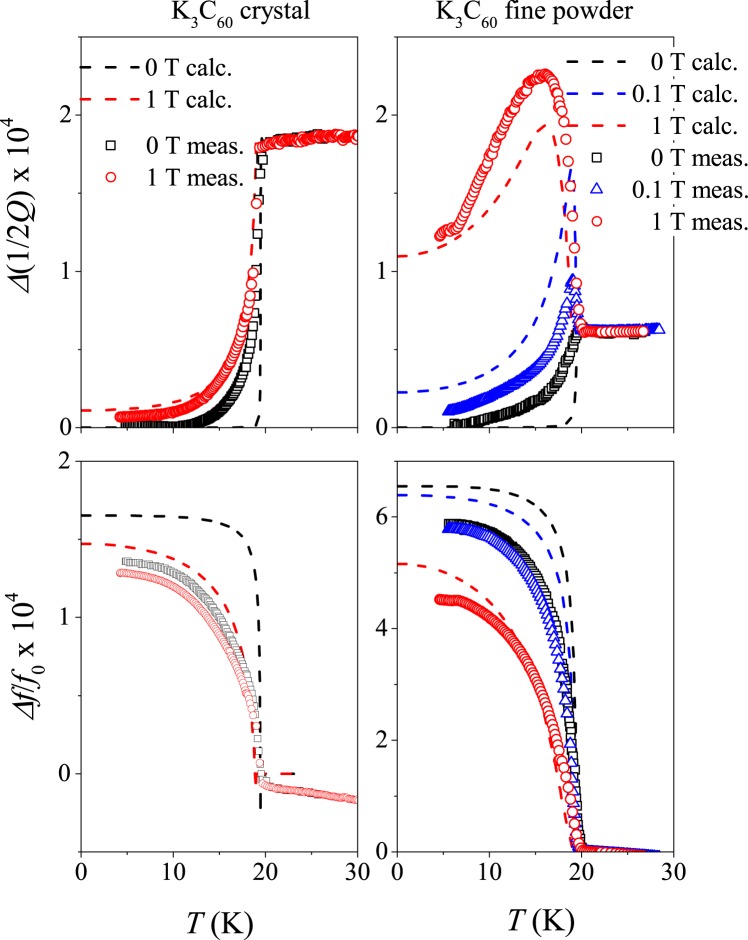
Comparison of measured and calculated cavity loss and shift parameters in the skin (left panels) and penetration limit (right panels). Calculation details are given in the text. Note that the calculated curves and the experimental data agree well for both sample types.

The left panels in Fig. 4. show the calculated and measured cavity loss and shift in 0 and 1 T magnetic fields for the single crystal sample. The calculation uses Eq. (3) with *κ*_p_ = *κ*_p,max_/20. Although this low *κ*_p_ induces a large *σ*_1_, there is no visible peak in the cavity loss in this case when excitation is limited to the surface. We discuss in detail in the Supplementary Material that the calculated cavity loss and shift are insensitive to the value of *κ*_p_ in this limit. Clearly, the experimental data for the K_3_C_60_ crystal match well the calculations.

A suitable $\nu =5.1\cdot {10}^{-4}$ was chosen to match the calculation to the experiment. We find that for both the calculation and experiment, the cavity loss parameter drops rapidly below *T*_c_, although *σ*_1_/*σ*_n_ is around unity due to the vortex motion. This effect is due to the development of a significant *σ*_2_/*σ*_n_ ~ 100, which limits the penetration of microwaves into the sample and thus reduces the loss. This means that the microwave surface impedance measurement is not capable of providing information about *σ*_1_ in the presence of vortex motion. We note that the experimental curves do not show such a rapid change as a function of temperature as the calculation. This may be related to the finite size and surface roughness of the single crystal sample.

Second, we discuss the opposite limit, when the microwave field penetrates into the sample (known as the penetration limit), the cavity measurables depend differently on the sample parameters. It was shown^63^ for a sphere with radius, *a*:

4 $$\frac{{\rm{\Delta }}f}{{f}_{0}}-{\rm{i}}{\rm{\Delta }}(\frac{1}{2Q})=-\,\gamma \tilde{\alpha },$$

5 $$\tilde{\alpha }=-\,\frac{3}{2}(1-\frac{3}{{a}^{2}{\tilde{k}}^{2}}+\frac{3}{a\tilde{k}}\,\cot (a\tilde{k})),$$

where $\tilde{k}=\tilde{n}\frac{\omega }{c}$ is the complex wavenumber, with $\tilde{n}=\sqrt{{\rm{i}}\tilde{\sigma }/{\varepsilon }_{0}\omega }$ being the complex index of refraction. The dimensionless *γ* is a sample volume dependent constant.

The right panels in Fig. 4, show the measured cavity loss and shift data for the fine powder sample together with a fit according to Eq. (5). To obtain these fits, we fixed the transport and magnetic parameters (*ρ*_n_, *ξ*_0_, *λ*_0_) of K_3_C_60_ to the respective mean values as given in Table 1. We assumed that the sample consists of spheres with a uniform diameter, *a*. The zero magnetic field data depends only on *γ* and *a* when the other parameters, *δ*_n_ and *λ*, are fixed. A fit to the *B* = 0 data yields $\gamma =\mathrm{5.5(2)}\cdot {10}^{-4}$, and *a* = 6.2(2) *μ*m. We then proceed to fit the magnetic field dependent data with *κ*_p_ as the only free parameter and we obtain ${\kappa }_{{\rm{p}}}=\mathrm{1.0(1)}\cdot {10}^{3}$ N/m^2^, which is about *κ*_p,max_/20. As shown in Fig. 4, the calculation agrees well with the experimental data. The presence of a finite microwave absorption at *B* = 1 T down to the lowest temperature appears to be surprising; its origin is therefore discussed qualitatively. Although the penetration depth due to superconductivity becomes smaller than the particle size, it is accompanied by a large (*σ*_1_/*σ*_n_ ≈ 30) conductivity due to the vortex motion. It means that a substantial microwave absorption occurs on the surface of the sample. The important observation is that the loss remains proportional to *σ*_1_ in this case, although its effect is reduced. This is further elaborated in the last part of the Supplementary Material.

Somewhat better fits could be obtained when letting *λ*, *ρ*_n_, and *B*_c2_ differ from the mean literature values. In addition, Eq. (5) is valid for spheres only, it thus fixes the ratio between the real and imaginary parts (cavity loss and shift). A different particle shape or particle size distribution would allow for a different scaling factor for the loss and shift data which could also improve the fits. Although improved fits could be attained, we believe that the simplest model explains well the experimental observation of an enhanced microwave absorption. In addition it allows to determine an effective pinning force constant, which is an important parameter to describe the electrodynamics of type-II superconductors. However, we note that *κ*_p_ determined herein may overestimate the bulk pinning force constant; it is known that the presence of a substantial surface-volume ratio may give rise to additional vortex pinning^58,59^, with a strength that is difficult to estimate.

## Summary

We demonstrated that moderate magnetic fields, which are small compared to the upper critical field, induces a large microwave absorption in fine powders of type II superconductors, like MgB_2_ and K_3_C_60_. The effect is absent for samples containing larger grains or compacted powder pellets. The Bardeen–Stephen model of flux-flow predicts that the real part of the AC conductivity can be enhanced in the microwave range, but this effect has not been observed. We analyze the conductivity using the Coffey–Clem theory which also accounts for vortex pinning effects. It is applied to calculate the microwave properties for two kinds of samples: when the electromagnetic field penetration is limited to the surface (skin limit) or when it fully penetrates into the fine grain samples (penetration limit). We show that microwave absorption in the skin limit is little affected by the vortex-motion enhanced *σ*_1_ but in the penetration limit, the effect is clearly observable. A quantitative analysis for K_3_C_60_ yields the vortex pinning force constant that can be hardly determined by other means. Our observation allowed us to explain long-standing microwave anomalies in superconductors^30,31^ and it may lead to pertinent applications in microwave communication techniques.

## Electronic supplementary material

Supplementary Information
