# Supplementary material for: Giant microwave absorption in fine powders of superconductors
Source: Sci Rep. 2018 Jul 31;8:11480. doi: 10.1038/s41598-018-29750-7 (PMC6068108; doi:10.1038/s41598-018-29750-7)
Supplement: Supplementary file 1 — Supplementary Information [file 41598_2018_29750_MOESM1_ESM.pdf]

Supplementary Material for: Giant microwave absorption in fine powders of superconductors

G. Csősz,[1] B. G. Márkus,[1] A. Jánossy,[1] N. M. Nemes,[2] F. Murányi,[3] G. Klupp,[4] K. Kamarás,[4] V. G. Kogan,[5] S. L. Bud'ko,[5] P. C. Canfield,[5] and F. Simon[1,*]

[1]*Department of Physics, Budapest University of Technology and Economics and MTA-BME Lendület Spintronics Research Group (PROSPIN), POBox 91, H-1521 Budapest, Hungary*

[2]*GFMC, Unidad Asociada ICMM-CSIC "Laboratorio de Heteroestructuras con Aplicacion en Espintronica", Departamento de Fisica de Materiales Universidad Complutense de Madrid, 28040 Madrid, Spain*

[3]*Mettler-Toledo GmbH, Heuwinkelstrasse 3., CH-8606 Nänikon, Switzerland*

[4]*Institute for Solid State Physics and Optics, Wigner Research Centre for Physics, Hungarian Academy of Sciences, P.O. Box 49, H-1525 Budapest, Hungary*

[5]*Ames Laboratory, U.S. Department of Energy and Department of Physics and Astronomy, Iowa State University, Ames, Iowa 50011, USA*

I. THE SUM RULE AND COMPLEX CONDUCTIVITY IN SUPERCONDUCTORS

For usual metals, the sum rule states[1]:

$$\frac{2}{\pi}\int_0^\infty \sigma_1(\omega)\,\mathrm{d}\omega = \frac{ne^2}{m^*} = \epsilon_0\Omega_{\mathrm{pl}}^2, \tag{1}$$

where n is the charge carrier density, e is the elementary charge, m^* is the effective mass, Ω_{pl} is the plasma frequency, and ϵ_0 is the vacuum dielectric constant. This is satisfied for the AC Drude model, where σ_1 and σ_2 read:

$$\sigma_1 = \frac{ne^2\tau}{m^*}\frac{1}{1+\omega^2\tau^2}, \tag{2}$$

$$\sigma_2 = \frac{ne^2\tau}{m^*}\frac{\omega\tau}{1+\omega^2\tau^2}. \tag{3}$$

For superconductors, the Ferrell–Glover–Tinkham (FGT) theory states that the sum rule[2,3] is still obeyed, i.e. the spectral weight is only *re-arranged* due to superconductivity but the value of the integral is retained.

In the following, we discuss the relevance of the sum rule in different superconductor regimes. In a type II superconductor, $\lambda \gg (\xi_0, \ell)$,where ξ_0 is the coherence length and ℓ is the mean free path, therefore local electrodynamics can be used. For such a case, $\sigma_1(\omega) = 0$ below the gap frequency, $\omega_{\mathrm{g}} = \frac{2\Delta}{\hbar}$ except for the delta function at $\omega = 0$. The clean limit is defined as $\xi_0 \ll \ell$, which is equivalent to $\omega_{\mathrm{g}} \gg 1/\tau$, since $\xi_0 = \hbar v_{\mathrm{F}}/\pi\Delta$. In this case, all the oscillator strength appears in the $\sigma_1 \propto \delta(\omega)$ function as:

$$\sigma_{1,\mathrm{clean}} = \frac{ne^2}{m^*}\delta(\omega) = \frac{\pi}{2\mu_0\lambda_{\mathrm{L}}^2}\delta(\omega), \tag{4}$$

$$\sigma_{2,\mathrm{clean}} = \frac{1}{\mu_0\omega\lambda_{\mathrm{L}}^2}, \tag{5}$$

where λ_{L} is the London penetration depth, related to the charge carrier concentration as: $\lambda_{\mathrm{L}} = \sqrt{\frac{m^*}{\mu_0 ne^2}}$ and also $\lambda_{\mathrm{L}} = c/\Omega_{\mathrm{pl}}$ (where c is the speed of light). Note that $\sigma_{1,\mathrm{clean}}$ clearly satisfies the sum rule.

In the dirty limit, $\xi_0 \gg \ell$ (which is the case in K_3C_{60}, given that $\xi_0 \approx 3$ nm and $\ell = 1$ nm)[4], we have $\omega_{\mathrm{g}} \ll 1/\tau$. Then, the oscillator strength in the delta function is reduced by approximately $\omega_{\mathrm{g}}\tau$ (which is smaller than 1). Pippard's aproximate expression for the penetration depth, λ, in the dirty limit

$$\lambda = \lambda_{\mathrm{L}}\sqrt{1+\frac{\xi_0}{\ell}}, \tag{6}$$

leads to the same result:

$$\sigma_{1,\mathrm{dirty}}(\omega=0) = \frac{\lambda_{\mathrm{L}}^2}{\lambda^2}\sigma_{1,\mathrm{clean}}(\omega=0) \approx \frac{\ell}{\xi}\sigma_{1,\mathrm{clean}}(\omega=0) = \omega_{\mathrm{g}}\tau\sigma_{1,\mathrm{clean}}(\omega=0). \tag{7}$$

A theoretical consideration of the charge carriers and effective mass in K_3C_{60} gave a clean limit result as $\lambda_{\mathrm{L}} = 160$ nm (Ref. 4) which is about a factor 3 smaller than the experimental value of $\lambda \sim 400$ nm. This means that the delta function strength is reduced by about a factor 10 as compared to the clean limit case.

In principle, one has two further regimes for type I superconductors, which are however not relevant for the present discussion. When $\xi_0 \ll \ell$, σ_1 and σ_2 have similar form as in the clean limit. When $\xi_0 \gg \ell$, one must use non-local electrodynamics, which also leads to a delta function with a reduced spectral weight.

II. FREQUENCY AND TEMPERATURE DEPENDENCE OF THE AC CONDUCTIVITY IN SUPERCONDUCTORS

The description of the AC conductivity was given by Mattis and Bardeen based on BCS theory in 1958.[5] The expressions for σ_1 and σ_2 are:

$$\frac{\sigma_1}{\sigma_{\mathrm{n}}} = \frac{2}{\hbar\omega}\int_\Delta^\infty \frac{[f(\varepsilon)-f(\varepsilon+\hbar\omega)]\left(\varepsilon^2+\Delta^2+\hbar\omega\varepsilon\right)}{\left(\varepsilon^2-\Delta^2\right)^{1/2}\left[(\varepsilon+\hbar\omega)^2-\Delta^2\right]}\mathrm{d}\varepsilon + \frac{1}{\hbar\omega}\int_{\Delta-\hbar\omega}^{-\Delta} \frac{[1-2f(\varepsilon+\hbar\omega)]\left(\varepsilon^2+\Delta^2+\hbar\omega\varepsilon\right)}{\left(\varepsilon^2-\Delta^2\right)^{1/2}\left[(\varepsilon+\hbar\omega)^2-\Delta^2\right]}\mathrm{d}\varepsilon, \tag{8}$$

$$\frac{\sigma_2}{\sigma_{\mathrm{n}}} = \frac{1}{\hbar\omega}\int_{\Delta-\hbar\omega,-\Delta}^{\Delta} \frac{[1-2f(\varepsilon+\hbar\omega)]\left(\varepsilon^2+\Delta^2+\hbar\omega\varepsilon\right)}{\left(\Delta^2-\varepsilon^2\right)^{1/2}\left[(\varepsilon+\hbar\omega)^2-\Delta^2\right]}\mathrm{d}\varepsilon, \tag{9}$$

where f is the usual Fermi-Dirac distribution function, and the lower limit of the integral in (9) for $\hbar\omega > 2\Delta$ is $-\Delta$.

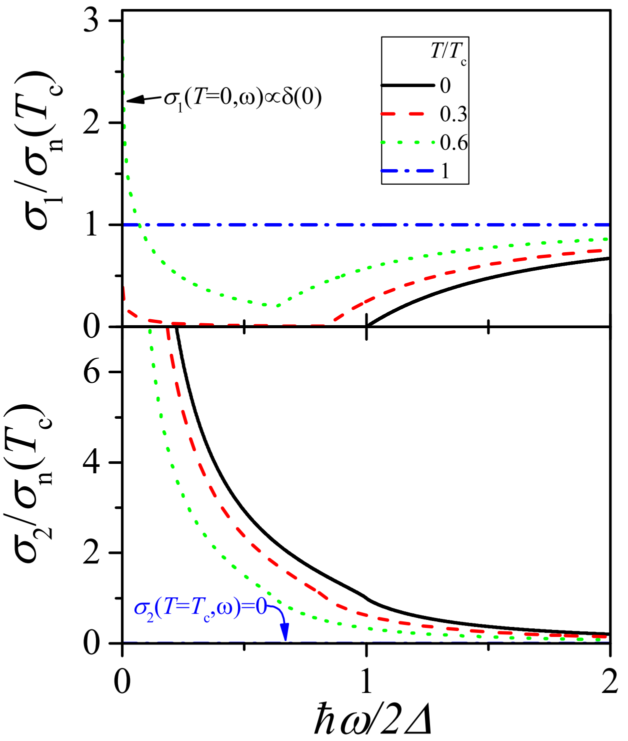

FIG. 1: Frequency dependence of the real and imaginary part of the conductivity calculated according to Mattis–Bardeen equations.

In Fig. 1., we show the real and imaginary parts of the AC conductivity as a function of frequency at some temperatures and $B = 0$. Note that σ_2 vanishes at $T = T_c$ and that σ_1 becomes a delta function, $\delta(\omega)$, at $T = 0$ and $\omega = 0$ with a spectral weight which compensates for the oscillator strength which is missing at finite frequencies (this is also known as the sum rule for superconductors[6]). The zero frequency conductivity in the superconducting range therefore equals to roughly: $\sigma_1(\omega) \approx \delta(\omega)\sigma_n\frac{2\Delta}{\hbar}$.

III. DETAILS OF SAMPLE PREPARATION AND MICROWAVE MEASUREMENT

Preparation of K_3C_{60} proceeds from high purity C_{60} (purity $> 99.9\,\%$, "super gold grade", Hoechsts) and K (Sigma-Aldrich) in stoichiometric amounts. It is heated to 350 °C in a closed steel capsule for typically 3 days in inert atmosphere followed by grinding of the sample and a final, 1 week-long, heat treatment under the same temperature conditions[7]. The sample was repeatedly ground followed by additional heat treatment to enable a homogeneous doping. Characterization was performed using IR spectroscopy, powder X-ray diffractometry, electron spin resonance spectroscopy and SQUID magnetometry.

The starting C_{60} crystal sample was grown with the gradient sublimation technique[8] which results in fullerene single crystals with the size from a few 100 microns up to 2 millimeters. The doping then proceeded along the conventional vapor phase doping. The superconducting volume fraction of the K_3C_{60} crystal was characterized by SQUID magnetometry and reported in Ref. 9. The zero-field cooled magnetization reveals[9], by field exclusion, a superconducting volume fraction close to 100 %. Note that this method can not reveal core-shell-like arrangements of cores of non-superconducting impurities shielded by superconducting shells. This, however, would not affect the present studies which are also limited to the surface of the K_3C_{60} crystal.

In the microwave measurements, we set $\Delta f/f_0 = 0$ at $T = T_c$ as Δf is affected by the quartz tube itself and the amount of helium in the cryostat, i.e. reference measurements do not give a reproducible result. This effect also influences the analysis of the data: an additional fitting parameter, the cavity shift value at $T = T_c$, needs to be introduced. Its value is $+6.7(2) \times 10^{-6}$, i.e. this value is subtracted from the calculated shift values which returns $\Delta f/f_0 = 0$ at $T = T_c$. This extra parameter merely shifts the $\Delta f/f_0$ curves.

IV. DETAILS OF THE EXPERIMENTAL SETUP

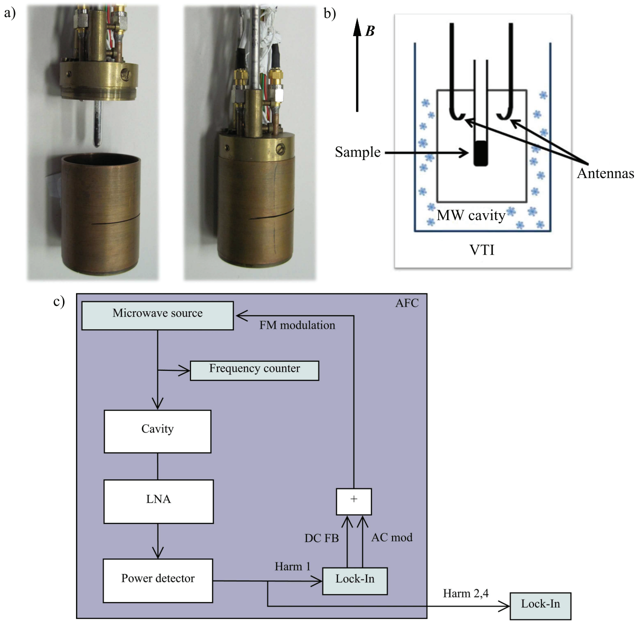

FIG. 2: a) Photograph of the probehead with the copper cavity removed such that the sample in the quartz tube is visible (left) and fully assembled (right). b) Schematics of the probehead indicating the sample, coupling microwave antennas, direction of the magnetic field, and also the surrounding VTI. c) Block diagram of the experiment which follows that in Ref. 10.

We measured the microwave conductivity using a low temperature probehead shown in Fig. 2a. It consists of the TE011 cylindrical copper cavity with the sample in its center, where the electric field has a node and the microwave magnetic field is maximal. Fig. 2b. shows the schematics of the cavity including the coupling microwave antenna and the direction of the DC magnetic field (parallel to the cavity

axis). The cavity is embedded inside the VTI. Fig. 2c. shows the schematics of the Q and f_0 measurement. It follows Ref. 10. The frequency of a microwave source (HP83752A) is locked to the cavity using a home-built automatic frequency control (AFC) circuit. The AFC is based on the frequency modulation of the source output frequency with about 20 kHz using a harmonic output of a lock-in (AC mod). The cavity responds with an amplitude modulated output signal, whose 20 kHz component is sign-sensitive to whether the frequency of the source is below or above f_0. A phase sensitive detection of this signal using a lock-in amplifier (Stanford Research SR830) results in a low frequency feedback signal (DC FB) which is superimposed to the modulating signal and forms the FM driving of the source. The frequency of the source is measured with a microwave counter (EIP Model 25B). It was shown in Ref. 10 that the 2nd and 4th harmonics of 20 kHz can be used to determine the Q factor as essentially these are proportional to the curvature of the Lorentzian resonance profile of the cavity. When implemented properly, this AC method provides Q values with a relatively low error as compared to more conventional frequency swept methods.

V. ADDITIONAL EXPERIMENTAL DATA

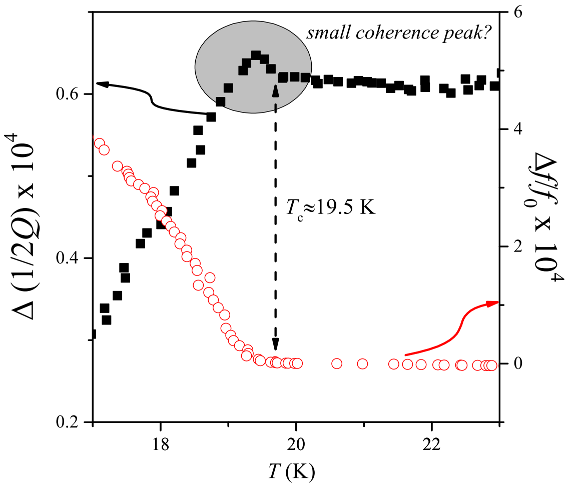

FIG. 3: Cavity loss and shift as a function of temperature at the Earth's magnetic field. These are the same data shown in the main text, however with a magnified scale. A small peak, which was tentatively assigned to superconducting coherence effects, is observed immediately below T_c.

In Fig. 3., we show the cavity loss and shift as a function of temperature in zero magnetic field. The data are zoomed to the vicinity of the critical temperature. A small coherence peak is clearly observed immediately below T_c. We are aware that this small coherence peak was observed in K_3C_{60} and also in Rb_3C_{60} ($T_c = 28$ K) back in 1994 but its observation remained unpublished[11]. In principle, a coherence peak is expected in the microwave conductivity as it is predicted by the Mattis–Bardeen theory and similar data were obtained in Ref. 12 for conventional weak-coupled superconductors. However, K_3C_{60} is a strong-coupled superconductor[4], therefore the suppression of the conductivity coherence peak is not surprising.

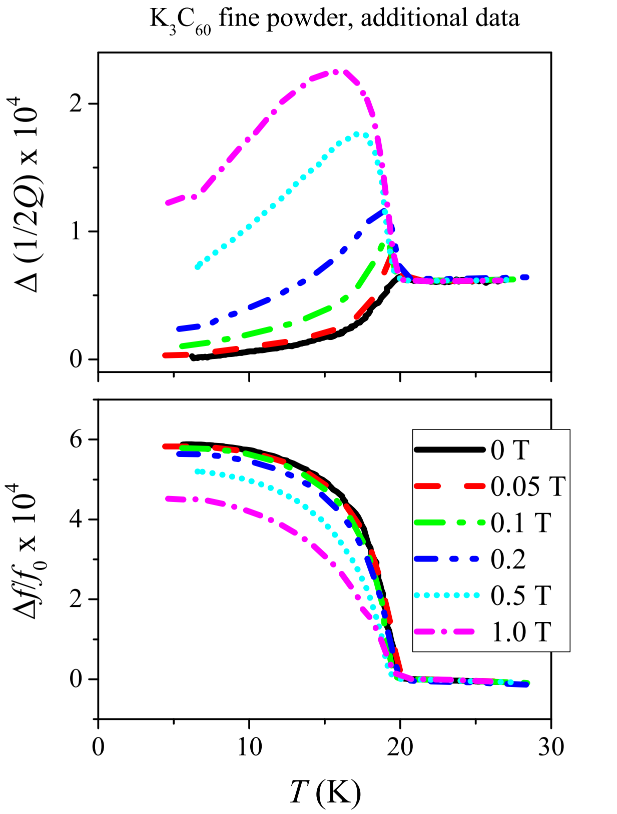

FIG. 4: Additional experimental data on the fine powder K_3C_{60} sample. The data at 0, 0.1, and 1 T are the same as in the main text.

In Fig. 4., we show additional magnetic field dependent cavity loss and shift data for the fine powder K_3C_{60} sample. The data at at 0, 0.1, and 1 T are identical to that shown in the main text.

VI. THE COFFEY–CLEM THEORY

The Coffey–Clem theory[13–18] describes the high frequency electrodynamic response of type-II-superconductors in the mixed state in external static magnetic field. In this calculation the static ($\mathbf{B}_0$) and the *rf* magnetic field are parallel and the static field is much greater than the amplitude of the *rf* field. This geometry matches our experimental setup. The corresponding *rf* electric field is responsible for the vortex movement and is perpendicular to the static magnetic field. The theory also assumes a uniform vortex density distribution.

To estimate the complex AC conductivity, the two-fluid equation $\mathbf{J} = \mathbf{J}_n + \mathbf{J}_s$ is used, where $\mathbf{J}_n$ is the normal current density, and $\mathbf{J}_s$ is supercurrent density, which is caused by the vortex dynamics. The current density is characterized by

the complex penetration depth, which is defined as follows:

$$\mathbf{J} = \widetilde{\sigma}\mathbf{E}, \tag{10}$$

$$\widetilde{\sigma} = \frac{\mathrm{i}}{\mu_0 \omega \widetilde{\lambda}^2}. \tag{11}$$

The quantity $\widetilde{\lambda}$ is determined from the current densities as follows. The two types of current density are obtained from $\mathbf{J}_\mathrm{n} = \sigma_\mathrm{nf}\mathbf{E}$ and the London equations:

$$\nabla \times \mathbf{J}_\mathrm{s} = -\frac{1}{\mu_0 \lambda^2}\left(\mathbf{B} - \mathbf{B}_\mathrm{v}\right), \tag{12}$$

where $\mathbf{B} = \mathbf{B}_0 + \mathbf{B}_\mathrm{rf}$, $\mathbf{B}_\mathrm{v} = n\Phi_0\widehat{\mathbf{B}}_0$ is the total magnetic field in the vortices, which is the sum of the external DC field ($\mathbf{B}_0$) and the vortex-motion-induced magnetic field. $\widehat{\mathbf{B}}_0$ is the direction of the DC magnetic field, n denotes the local area density of vortices. These relationships together with Ampere's law yield:

$$\nabla^2\mathbf{B} = \mu_0\sigma_\mathrm{nf}\dot{\mathbf{B}} + \frac{1}{\lambda^2}\left(\mathbf{B} - \mathbf{B}_\mathrm{v}\right). \tag{13}$$

The last term can be obtained from the vortex equation of motion:

$$\mu\ddot{\mathbf{u}} + \eta\dot{\mathbf{u}} + \kappa_p\mathbf{u} = \mathbf{J} \times \Phi_0\widehat{\mathbf{B}}_0, \tag{14}$$

where $\mathbf{u}$ is the vortex displacement from its equilibrium pinning site, $\mu\left(T\right) = \epsilon_0\Phi_0 B_{c2}\left(T\right)$ is the inertial mass per unit length of vortex, η is the viscous drag coefficient in the absence of flux creep and κ_p is the restoring force constant of a pinning potential. All these parameters depend on temperature.

The viscous drag coefficient is directly determined by the other parameters as[19–21]:

$$\eta\left(T\right) = \frac{\Phi_0 B_{c2}\left(T\right)}{\rho_\mathrm{n}\left(T\right)}. \tag{15}$$

(16)

However, only the maximum value for the pinning force constant can be estimated[19–21]. It is obtained for a perfect pinning center, which is a hollow core with radius ξ:

$$\kappa_\mathrm{p,max} = \frac{B_{c1}B_{c2}}{2\mu_0} / \ln\left(\frac{\lambda}{\xi}\right), \tag{17}$$

where μ_0 is the vacuum permeability. In a realistic case, κ_p can be substantially smaller than $\kappa_\mathrm{p,max}$ depending on the quality of the pinning centers.

For a harmonic *rf* excitation, Eq. (14) can be rewritten with the help of the complex dynamic vortex mobility $\widetilde{\mu}_\mathrm{v} = \left(-\mathrm{i}\omega\mu + \eta + \mathrm{i}\kappa_p/\omega\right)^{-1}$ as:

$$\mathbf{u} = -\frac{\widetilde{\mu}_\mathrm{v}}{\mathrm{i}\omega}\mathbf{J} \times \Phi_0\widehat{\mathbf{B}}_0. \tag{18}$$

Note the distinction between μ (inertial mass per unit length of vortex), μ_v (complex dynamic vortex mobility), and μ_0 (permeability of the vacuum).

When $\mathbf{J}$ and $\widehat{\mathbf{B}}_0$ are perpendicular, and the vortex-motion-induced electric field is $\mathbf{E}_\mathrm{v} = \mathbf{B}_\mathrm{v} \times \dot{\mathbf{u}}$ which obeys $\frac{\partial \mathbf{B}_\mathrm{v}}{\partial t} = -\nabla \times \mathbf{E}_\mathrm{v}$:

$$\mathbf{B}_\mathrm{v} = \mathbf{B}_0 - \nabla \times \left(\mathbf{B}_0 \times \mathbf{u}\right). \tag{19}$$

Considering the vector calculus identities ($\nabla \times (\nabla \times \mathbf{B}) = \nabla(\nabla\mathbf{B}) - \nabla^2\mathbf{B}$) and Faraday's law with harmonic *rf* excitation ($-\nabla \times \mathbf{E} = \dot{\mathbf{B}} = -\mathrm{i}\omega\mathbf{B}$) with the help of equation (19), equation (13) can be rewritten:

$$-\nabla \times (\nabla \times \mathbf{B}) = -\mu_0\sigma_\mathrm{nf}\nabla \times \mathbf{E} + \frac{1}{\lambda^2}\left(\frac{1}{\mathrm{i}\omega}\nabla \times \mathbf{E} + \nabla \times \left(\mathbf{B}_0 \times \mathbf{u}\right)\right). \tag{20}$$

The components of this equation can be integrated with respect to the space variable:

$$\mu_0\mathbf{J} = \mu_0\sigma_\mathrm{nf}\mathbf{E} + \frac{1}{\lambda^2}\left(-\frac{1}{\mathrm{i}\omega}\mathbf{E} + \frac{\Phi_0 B_0\widetilde{\mu}_\mathrm{v}}{\mathrm{i}\omega}\mathbf{J}\right), \tag{21}$$

$$\mathbf{J} = \frac{\mu_0\sigma_\mathrm{nf} - \frac{1}{\lambda^2\mathrm{i}\omega}}{\mu_0 - \frac{\Phi_0 B_0\widetilde{\mu}_\mathrm{v}}{\mathrm{i}\omega\lambda^2}}\mathbf{E}, \tag{22}$$

$$\widetilde{\lambda}^2 = \frac{\lambda^2 + (\mathrm{i}/2)\widetilde{\delta}^2_\mathrm{vc}}{1 - 2\mathrm{i}\lambda^2/\widetilde{\delta}^2_\mathrm{nf}}. \tag{23}$$

The latter quantity is the so-called complex penetration depth. In these equations, $\widetilde{\delta}_\mathrm{nf}$ is the normal fluid skin depth, and $\widetilde{\delta}^2_\mathrm{vc} = \frac{2B_0\Phi_0\widetilde{\mu}_\mathrm{v}}{\mu_0\omega}$ is the complex effective skin depth. The latter quantity is zero for $B = 0$ and it becomes finite in the mixed state when vortex motion is present. Note that for $B = 0$, i.e. $\widetilde{\delta}^2_\mathrm{vc} = 0$, $\widetilde{\lambda}^2$ returns λ^2 and $\mathrm{i}\delta^2_\mathrm{n}/2$ at $T = 0$ and $T = T_\mathrm{c}$, respectively.

The temperature and magnetic field dependence of the

transport and magnetic parameters are:

$$B_{c2}(T) = B_{c2}(0)\frac{1-t^2}{1+t^2}, \tag{24}$$

$$\lambda(T,B) = \frac{\lambda(0,0)}{\sqrt{(1-b(T))(1-t^4)}}, \tag{25}$$

$$\widetilde{\delta}_{\mathrm{nf}}^2(T,B) = \delta_{\mathrm{n}}^2/f(T,B), \tag{26}$$

$$f(T,B) = 1-\left(1-t^4\right)(1-b(T)), \tag{27}$$

$$\kappa_{\mathrm{p}}(T) = \kappa_{\mathrm{p}}(0)\left(1-t^2\right)^2, \tag{28}$$

where $t = T/T_{\mathrm{c}}$ and $b(T) = B/B_{c2}(T)$ are the reduced temperature and magnetic field, respectively.

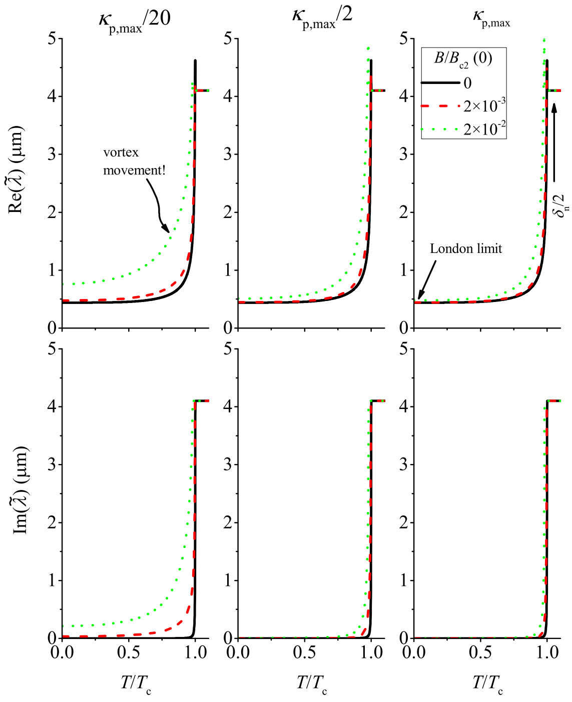

FIG. 5: Temperature dependence of the real and imaginary parts of $\widetilde{\lambda}$ for various magnetic fields and κ_{p} values. Note that Re $\left(\widetilde{\lambda}\right)$ returns the London penetration depth at zero magnetic field and also shows the effect of vortex movement for a finite B. In the normal state, it returns $\delta_{\mathrm{n}}/2$ as expected.

It is informative to show the temperature dependence $\widetilde{\lambda}$, which correspond to the presented $\widetilde{\sigma}$ in the main text. This is shown for various magnetic fields and κ_{p} values in Fig. 5. In zero magnetic field, Re $\left(\widetilde{\lambda}\right)$ drops rapidly from $\delta_{\mathrm{n}}/2$ at $T = T_{\mathrm{c}}$ to the London penetration depth toward $T = 0$. However, for finite magnetic fields and a smaller κ_{p}, Re $\left(\widetilde{\lambda}\right)$ remains closer to the normal state value down to about half of T_{c} due to the vortex induced conductivity. For small grain sizes, this allows for a significant microwave field penetration into the grains which leads to a sizeable microwave cavity loss such as we observe herein.

VII. ADDITIONAL DISCUSSION OF THE VORTEX MOTION IN EXTERNAL STATIC AND *RF* MAGNETIC FIELD

In the following discussion we assume that the complex conductivity is dominated by the flux flow. So we neglect the effect of vortex pinning and flux creep. In this case the vortex motion is retarded only by the viscous damping. So we can obtain the vortex motion in the following form[6]:

$$\dot{\mathbf{u}} = \frac{1}{\eta}\mathbf{J}\times\Phi_0\widehat{\mathbf{B}}_0. \tag{29}$$

The current density is calculated from Ohm's law in which the electric field is induced by the external *rf* magnetic field:

$$\nabla\times\mathbf{J}_{\mathrm{ac}} = -\frac{1}{\rho_{\mathrm{f}}}\dot{\mathbf{B}}_{\mathrm{ac}}, \tag{30}$$

where ρ_{f} is the flux-flow resistivity $\left(\rho_{\mathrm{f}} = \frac{B\Phi_0}{\eta}\right)$. For sphere samples the solution of the (30) equation is the following in cylindrical coordinates:

$$\mathbf{J}_{\mathrm{ac}} = -\frac{r}{2\rho_{\mathrm{f}}}\dot{\mathbf{B}}_{\mathrm{ac}}, \tag{31}$$

The directions of the vectors are shown in Fig. 6. Equation

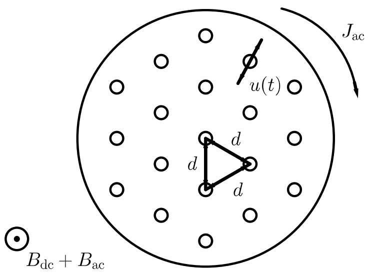

FIG. 6: Sphere superconductor in external static and *rf* magnetic field. The magnetic field is parallel to the z axis, and the induced current density is tangential. So the direction of the vortex motion is radial.

(29) can be rewritten with this result:

$$\dot{u} = -\frac{r}{2}\frac{\dot{B}_{\mathrm{ac}}}{B}. \tag{32}$$

This result is the same, if we assume that vortex motion is caused by the change of vortex density. The reason of this change is the small oscillation in the magnetic field (small so that only the size of the primitive cell of the vortex lattice varies). The only relevant length scale is the distance between the vortices, which is proportional to $\left(\frac{\Phi_0}{B}\right)^{1/2}$.[6] The motion of the vortices can only be radial due to the lattice symmetry. Thus the velocity of the vortices is equal to the time derivative of r, where:

$$r = n\left(\frac{\Phi_0}{B}\right)^{1/2}, \tag{33}$$

where n depends only on the vortex lattice structure. The time derivative of r is:

$$\dot{r} = \underbrace{-n\left(\frac{\Phi_0}{B}\right)^{1/2}}_{r} \frac{\dot{B}_{\mathrm{ac}}}{2B} = -\frac{r}{2}\frac{\dot{B}_{\mathrm{ac}}}{B}. \tag{34}$$

VIII. FREQUENCY DEPENDENCE OF THE CONDUCTIVITY IN THE PRESENCE OF VORTEX MOTION

It is assumed that conductivity can be written as a sum of two terms: $\sigma = \sigma_{\mathrm{nf}} + \sigma_{\mathrm{sf}}$, where σ_{nf} and σ_{sf} are the normal-fluid and superconductor contributions, respectively. At low temperatures, the normal fluid term $\left(\lambda^2 \ll \delta_{\mathrm{nf}}^2\right)$ can be neglected, thus Eq. (13) can be rewritten as follows:

$$\mu_0\lambda^2\dot{\mathbf{J}} + \frac{1}{\sigma_{\mathrm{v}}(\omega)}\mathbf{J} = \mathbf{E}. \tag{35}$$

Note, that this equation is very similar to the kinetic equation of electrons in the AC Drude model: $\frac{m}{ne^2}\dot{\mathbf{J}} + \frac{1}{\sigma_0}\mathbf{J} = \mathbf{E}$.

According to the CC theory, the value of σ_{v} can be written as[13]:

$$\sigma_{\mathrm{v}}(\omega) = \sigma_{\mathrm{f}}\left(1 + \frac{\kappa_p}{\mathrm{i}\omega\eta}\right), \tag{36}$$

where $\sigma_{\mathrm{f}} = \frac{B_{\mathrm{c2}}}{B}\sigma_{\mathrm{n}}$ is the flux-flow conductivity (which appears in the Bardeen–Stephen theory, i.e. it is without vortex pinning).

In the absence of vortex pinning centers, $\sigma_{\mathrm{v}} = \sigma_{\mathrm{f}}$ i.e. it is the inverse of the Bardeen–Stephen resistivity[22]. In this case σ_{v} does not depend on frequency, so the frequency-dependent conductivity has the same form as the conductivity in the Drude model, shown in Fig. 7.

$$\sigma(\omega) = \frac{\sigma_{\mathrm{f}}}{1 + \mathrm{i}\omega\mu_0\lambda^2\sigma_{\mathrm{f}}}. \tag{37}$$

Note that (37) satisfies the sum rule:

$$\frac{2}{\pi}\int_0^\infty \mathrm{Re}\,\sigma(\omega)\,\mathrm{d}\omega = \frac{1}{\pi}\int_{-\infty}^\infty \frac{\sigma_{\mathrm{f}}}{1 + \omega^2\left(\mu_0\lambda^2\sigma_f\right)^2}\mathrm{d}\omega = \frac{1}{\mu_0\lambda^2}, \tag{38}$$

where we exploited that $\mathrm{Re}\,\sigma(\omega)$ is even and the $\frac{1}{1+\omega^2\tau^2}$ function has one simple pole at i/τ.

The conductivity has a more complicated form according to the CC theory when $\kappa_{\mathrm{p}} \neq 0$:

$$\sigma_1(\omega) = \frac{\sigma_f\lambda_{\mathrm{C}}^4}{\left(\lambda_{\mathrm{C}}^2\mu_0\omega\lambda^2\sigma_f\right)^2 + \left(\lambda_{\mathrm{C}}^2 + \lambda^2\right)^2} + \frac{\pi}{2}\frac{\delta(\omega)}{\mu_0\left(\lambda^2 + \lambda_{\mathrm{C}}^2\right)}, \tag{39}$$

$$\sigma_2(\omega) = -\frac{\lambda_{\mathrm{C}}^4\mu_0^2\omega^2\lambda^2\sigma_f^2 + \lambda^2 + \lambda_{\mathrm{C}}^2}{\mu_0\omega\left(\left(\lambda_{\mathrm{C}}^2\mu_0\omega\lambda^2\sigma_f\right)^2 + \left(\lambda_{\mathrm{C}}^2 + \lambda^2\right)^2\right)} \tag{40}$$

where $\lambda_{\mathrm{C}}^2 = B\Phi_0/\mu_0\kappa_{\mathrm{p}}$ is the so-called Campbell penetration depth[23,24].

The CC theory provides the first term only in σ_1. The presence of the second term with the delta function was inserted in order to satisfy the sum rule and to maintain the Kramers–Kronig relation between σ_1 and σ_2. The second terms in $\sigma_{1,2}$ are due to the strongly pinned vortices, i.e. these appear as those in superconductors in the Meissner state. The spectral weight is distributed between the two terms in σ_1: as the pinning force is increased, the delta function dominates and eventually it returns the case of $B = 0$. In the other extreme, when κ_{p} tends to zero, the Bardeen–Stephen result is recovered. The full result is shown in Fig. 8. for a few κ_{p} values.

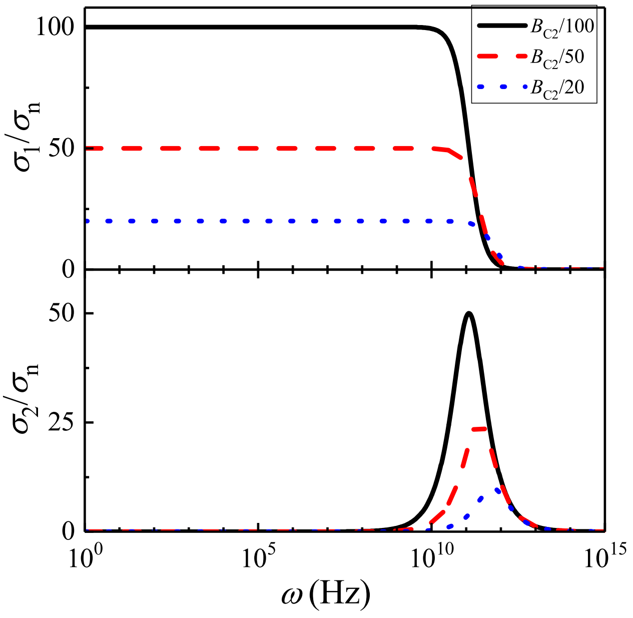

FIG. 7: Complex conductivity in different magnetic fields with no pinning centers. Note that when $B \to 0$ we recover the delta function in σ_1. Note, that the scale of the abscissa is logarithmic, as a result the lowering of the edge in the σ_1 data can be be hardly seen.

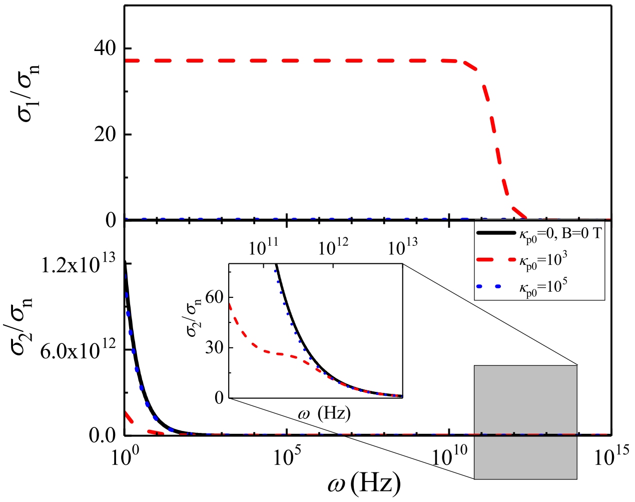

FIG. 8: Complex conductivity in finite magnetic field with different pinning forces; a new term, proportional to $1/\omega$, appears in σ_2. For the σ_1 data, the curves with large κ_{p} and $B = 0$ T values remain zero except for a delta function. Note that for a large pinning force, σ_1 and σ_2 have the same form as the conductivity in zero applied magnetic field. The scale of the abscissa is logarithmic. A zoom-in is shown for the grey shaded box in the insert.

IX. MICROWAVE CAVITY PERTURBATION IN THE SKIN LIMIT

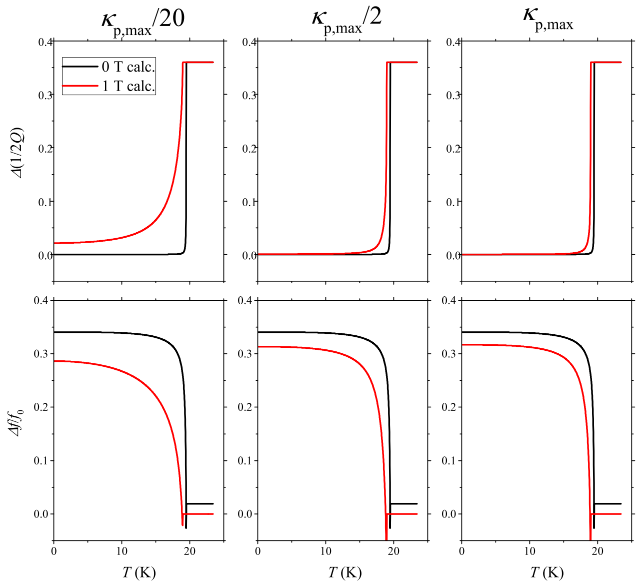

FIG. 9: Calculated cavity loss and shift calculated according to Eq. (41) using the σ_1 and σ_2 data shown in the main text at $f = 10\,\text{GHz}$. We used $\nu = 1$ in the calculation and assumed that the sample is in the node of the electric field inside a microwave cavity.

As discussed in the main text, when the penetration of microwaves is limited to the skin depth only, the microwave cavity loss and shift is expressed by the complex penetration depth $\widetilde{\lambda}$ as:

$$\frac{\Delta f}{f_0} - \mathrm{i}\Delta\left(\frac{1}{2Q}\right) = -\mathrm{i}\nu\mu_0\omega\sqrt{-\widetilde{\lambda}^2}, \tag{41}$$

where ν is called resonator constant[12].

Fig. 9. shows the cavity loss calculated according to Eq. (41) for various values of κ_p (the same σ_1 and σ_2 data as in the main text). Although σ_1 and σ_2 changes significantly for the various κ_p values, remarkably, in this limit the cavity loss and shift show little sensitivity to κ_p. As a result, surface impedance studies cannot determine the value of the pinning force constant with certainty.

Fig. 10. depicts the sensitivity of the cavity loss as a function of $\sigma_2/\sigma_\mathrm{n}$. Well below T_c, the latter quantity is about 100. At the same time, $\sigma_1/\sigma_\mathrm{n}$ remains around unity if the vortex motion is significant. However, in the skin limit regime, the cavity loss drops by about 2 orders of magnitude due to the significant σ_2, which fully prevents any meaningful measurement of σ_1 in surface impedance studies.

X. MICROWAVE CAVITY PERTURBATION IN THE PENETRATION LIMIT

We mentioned in the main text that for a small particle with diameter a the following relation holds between the

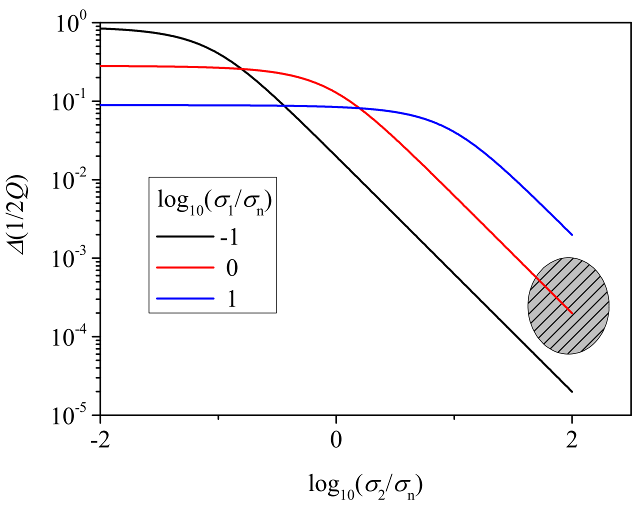

FIG. 10: Calculated cavity loss vs normalized σ_2 in logarithmic scale for different σ_1 values with the geometric factor, $\nu = 1$. The shaded area shows the experimentally relevant range of parameters.

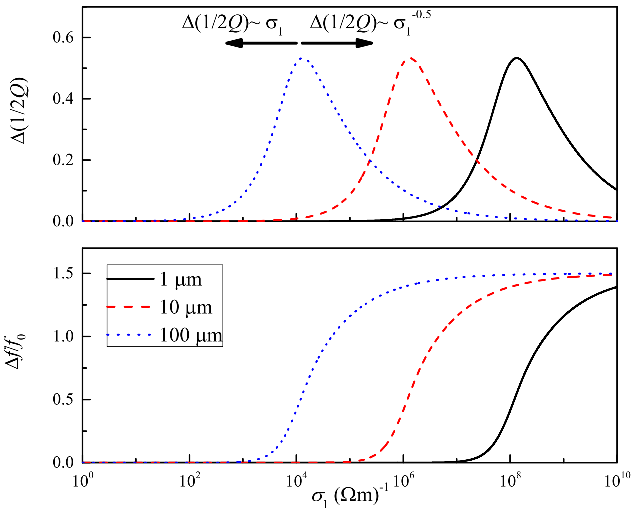

FIG. 11: Calculated cavity loss and shift vs. σ_1 when $\sigma_2 = 0$ for different sample sizes with Eq. (43) on a semilogarithmic plot using the geometrical constant $\gamma = 1$ at $f = 10\,\text{GHz}$.

cavity parameters and the conductivity[25]:

$$\frac{\Delta f}{f_0} - \mathrm{i}\Delta\left(\frac{1}{2Q}\right) = -\gamma\widetilde{\alpha}, \tag{42}$$

$$\widetilde{\alpha} = -\frac{3}{2}\left(1 - \frac{3}{a^2\widetilde{k}^2} + \frac{3}{a\widetilde{k}}\cot(a\widetilde{k})\right), \tag{43}$$

where $\widetilde{k} = \widetilde{n}\frac{\omega}{c}$ is the complex wavenumber with $\widetilde{n} = \sqrt{\frac{\mathrm{i}\widetilde{\sigma}}{\epsilon_0\omega}}$ being the complex index of refraction. Eq. (43) returns $\frac{\Delta f}{f_0} = 0$ and $\Delta\left(\frac{1}{2Q}\right) \propto \sigma$ for small values of σ as expected[26,27]. In the other extreme, it returns $\frac{\Delta f}{f_0} = -\gamma\frac{3}{2}$ and $\Delta\left(\frac{1}{2Q}\right) \propto 1/\sqrt{\sigma}$ for large values of σ, which is also the expected result[26,27].

Fig. 11. shows the calculated cavity loss and cavity shift as a function of σ_1 with $\sigma_2 = 0$ for various particle sizes as calculated with Eq. (43). This calculation demonstrates that the character of the loss vs σ_1 changes depending on the value of σ_1 from a $\Delta\left(\frac{1}{2Q}\right) \propto \sigma_1$ to a $\Delta\left(\frac{1}{2Q}\right) \propto 1/\sqrt{\sigma_1}$, where the characteristic value of the crossover is also particle size dependent. The crossover is accompanied by a change in the cavity shift.

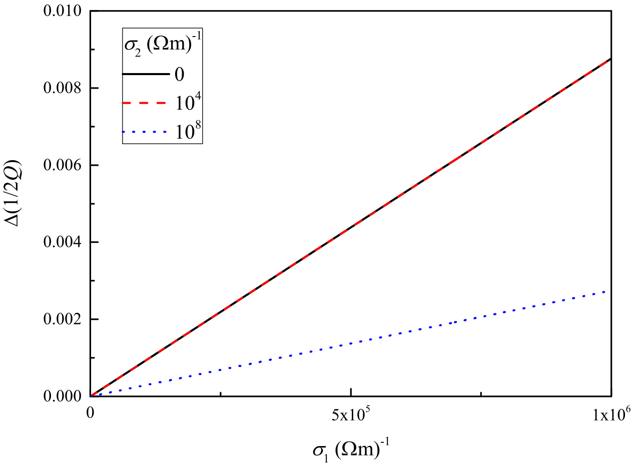

FIG. 12: Calculated cavity loss vs. σ_1 for different σ_2 values. The sample size is 1 μm. Cavity loss is in arbitrary units, i.e. multiplied by an arbitrary γ. Note that the loss becomes less sensitive to σ_1 for larger σ_2 values (dotted line) but the solid and dashed lines overlap.

Fig. 12. demonstrates that the cavity loss decreases for a larger value of σ_2. This is the reason why in the superconducting state, where σ_2 is finite, it is more difficult to detect the effect of the vortex motion induced finite σ_1 on the cavity loss. Nevertheless, the drop in the cavity loss even for a large $\sigma_2 = 10^8$ $1/\Omega$m is about a factor 3 as compared to the $2-3$ orders of magnitude cavity loss drop for the same σ_2 for a single crystal sample. The latter situation is shown in Fig. 10.

* Electronic address: f.simon@eik.bme.hu

[1] Kubo, R. Statistical-Mechanical Theory of Irreversible Processes. I. General Theory and Simple Applications to Magnetic and Conduction Problems. *Journal of the Physical Society of Japan* **12**, 570–586 (1957).

[2] Ferrell, R. A. & Glover, R. E. Conductivity of Superconducting Films: A Sum Rule. *Phys. Rev.* **109**, 1398–1399 (1958).

[3] Tinkham, M. & Ferrell, R. A. Determination of the Superconducting Skin Depth from the Energy Gap and Sum Rule. *Phys. Rev. Lett.* **2**, 331–333 (1959).

[4] Gunnarsson, O. Superconductivity in fullerides. *Rev. Mod. Phys.* **69**, 575–606 (1997).

[5] Mattis, D. C. & Bardeen, J. Theory of the Anomalous Skin Effect in Normal and Superconducting Metals. *Phys. Rev.* **111**, 412–417 (1958).

[6] Tinkham, M. *Introduction to superconductivity* (Krieger Publishing Company, 1975).

[7] Fleming, R. M. *et al.* Relation of structure and superconducting transition-temperature in A_3C_{60}. *Nature* **352**, 787–788 (1991).

[8] Dresselhaus, M. S., Dresselhaus, G. & Ecklund, P. C. *Science of Fullerenes and Carbon Nanotubes* (Academic Press, 1996).

[9] Nemes, N., Fischer, J. E., Baumgartner, G., Forró, L. & Jánossy, A. Conduction electron spin resonance in the superconducting state of K_3C_{60}. In Kuzmany, H. (ed.) *AIP Conference Proceedings 486*, 56–59 (1999).

[10] Nebendahl, B., Peligrad, D.-N., Pozek, M., Dulcic, A. & Mehring, M. An ac method for the precise measurement of Q-factor and resonance frequency of a microwave cavity. *Rev. Sci. Instrum.* **72**, 1876 (2001).

[11] Jánossy, A. & Legeza, O. private communications.

[12] Klein, O., Nicol, E. J., Holczer, K. & Grüner, G. Conductivity coherence factors in the conventional superconductors Nb and Pb. *Phys. Rev. B* **50**, 6307–6316 (1994).

[13] Coffey, M. W. & Clem, J. R. Unified Theory of Effects of Vortex Pinning and Flux Creep upon the rf Surface Impedance of Type-II Superconductors. *Phys. Rev. Lett.* **67**, 386–389 (1991).

[14] Coffey, M. W. & Clem, J. R. Theory of rf magnetic permeability of isotropic type-II superconductors in parallel field. *Phys. Rev. B* **45**, 9872–9881 (1992).

[15] Coffey, M. W. & Clem, J. R. Theory of rf magnetic permeability of type-II superconductors in slab geometry with an oblique applie static magnetic field. *Phys. Rev. B* **45**, 10527–10535 (1992).

[16] Coffey, M. W. & Clem, J. R. Coupled nonlinear electrodynamics of type-II superconductors in the mixed state. *Phys. Rev. B* **46**, 567–570 (1992).

[17] Coffey, M. W. & Clem, J. R. Theory of high-frequency linear response of isotropic type-II superconductors in the mixed state. *Phys. Rev. B* **46**, 11757–11764 (1992).

[18] Coffey, M. W. & Clem, J. R. Theory of microwave transmission and reflection in type-II superconductors in the mixed state. *Phys. Rev. B* **48**, 342–350 (1993).

[19] Anderson, P. Theory of flux creep in hard superconductors. *Phys. Rev. Lett.* **9**, 309–311 (1962).

[20] Wu, D.-H. & Sridhar, S. Pinning forces and lower critical fields in $YBa_2Cu_3O_y$ crystals: Temperature dependence and anisotropy. *Phys. Rev. Lett.* **65**, 2074–2077 (1990).

[21] Lévy, L.-P. *Magnetism and Superconductivity* (Springer-Verlag, Berlin, Heidelberg, 2000).

[22] Bardeen, J. & Stephen, M. J. Theory of the Motion of Vortices in Superconductors. *Phys. Rev.* **140**, A1197–A1207 (1965).

[23] Campbell, A. M. The response of pinned flux vortices to low-frequency fields. *Journal of Physics C: Solid State Physics* **2**, 1492 (1969).

[24] Campbell, A. M. The interaction distance between flux lines and pinning centres. *Journal of Physics C: Solid State Physics* **4**, 3186 (1971).

[25] Landau, L. D. & Lifschitz, E. M. *Electrodynamics of Continuous Media, Course of Theoretical Physics, Vol. 8* (Pergamon Press, Oxford, UK, 1984).

[26] Klein, O., Donovan, S., Dressel, M. & Grüner, G. Microwave cavity perturbation technique: Part I: Principles. *Int. J. Infr. Mill. Wav.* **14**, 2423–2457 (1993).

[27] Kitano, H. *et al.* Evidence for Insulating Behavior in the Electric Conduction of $NH_3K_3C_{60}$ Systems. *Phys. Rev. Lett.* **88**, 096401 (2002).
